# Printed unmanned aerial vehicles using paper-based electroactive polymer actuators and organic ion gel transistors

**DOI:** 10.1038/micronano.2016.32

**Published:** 2016-08-15

**Authors:** Gerd Grau, Elisha J. Frazier, Vivek Subramanian

**Affiliations:** 1 Department of Electrical Engineering and Computer Sciences, University of California, Berkeley, CA 94720-1770, USA

**Keywords:** electroactive polymer (EAP) actuators, inexpensive flight system integration, ion gel-gated organic thin film transistors (OTFTs), lightweight paper substrates, printed electronics, unmanned aerial vehicle

## Abstract

We combined lightweight and mechanically flexible printed transistors and actuators with a paper unmanned aerial vehicle (UAV) glider prototype to demonstrate electrically controlled glide path modification in a lightweight, disposable UAV system. The integration of lightweight and mechanically flexible electronics that is offered by printed electronics is uniquely attractive in this regard because it enables flight control in an inexpensive, disposable, and easily integrated system. Here, we demonstrate electroactive polymer (EAP) actuators that are directly printed into paper that act as steering elements for low cost, lightweight paper UAVs. We drive these actuators by using ion gel-gated organic thin film transistors (OTFTs) that are ideally suited as drive transistors for these actuators in terms of drive current and frequency requirements. By using a printing-based fabrication process on a paper glider, we are able to deliver an attractive path to the realization of inexpensive UAVs for ubiquitous sensing and monitoring flight applications.

## Introduction

Unmanned aerial vehicles (UAVs) are becoming increasingly ubiquitous for use in tasks ranging from information gathering to the delivery of goods. Simultaneously, the development of printed and flexible electronics has received much attention as a path toward the realization of ubiquitous electronics enabled by the inexpensive, large-area fabrication of electronic systems on flexible substrates such as plastic and paper^1,2^. These novel substrates are not only inexpensive but also superbly lightweight and mechanically flexible, thus making them ideal for applications such as flight. The combination of printed electronics with UAVs adds a new dimension to the range of applications of ubiquitous electronics that fully exploits the weight and flexibility advantages of printed electronics. If UAVs could be fully produced via printing, then inexpensive UAVs could be mass deployed for applications such as monitoring of environmental effects, including pollutants or wildfires, search and rescue missions or tactical security applications^3,4^. There are multiple ways in which such UAVs could be controlled. First, the flight path might be programmed before take-off, for example, if the UAV is to survey a particular fixed area. Second, the UAV might contain control circuits that allow it to react to the signals measured by its sensors, such as changing wind directions that require course corrections, or to follow signals of interest, such as detected wildfires. Third, the UAV might be remotely controlled by a wireless system^5^. Each of these solutions might be the method of choice for a particular application; however, the first two solutions are most promising to deploy large numbers of inexpensive UAVs. Biodegradable substrates such as paper are especially interesting for disposable UAV systems. Most of the electrical components required for such a system have already been demonstrated on flexible substrates; these components include sensors, batteries, solar cells, transistors, passive components, and antennas^6–17^. The key remaining piece is the realization of mechanical actuation with a form factor and weight that are suitable for UAV integration. UAVs require mechanical components for propulsion and steering. Active propulsion is challenging to implement by printing. Generally, more complex geometries are required compared with the two-dimensional rudders used for steering. In addition, active propulsion generally requires a larger actuation speed than steering does. In the future, active propulsion might be implemented in a biomimetic fashion by printing flexible morphing wings. This requires not only high-performance actuators but also a detailed understanding of the aerodynamics of such wings. Until then, active propulsion will not be required for applications in which gliders can be released at high altitudes (for example, from carrier aircraft). Mechanical steering elements must be printed to create fully printed, functional UAVs. Here, we demonstrate steering elements based on printed electroactive polymer (EAP) actuators on paper. Infusing EAP into paper enables the paper that acts as the structural material for the UAV to become electromechanically active. We demonstrate simple flaps that can control the flight direction of a paper UAV, thus allowing us to demonstrate the use of printed actuators to control UAV flight for the first time. The entire paper glider UAV, including actuators, batteries, interconnects, and the paper glider itself, weighs <8 g. This is a compelling demonstration of the advantages of exploiting lightweight and flexible electronics for UAVs.

**Figure 1:**
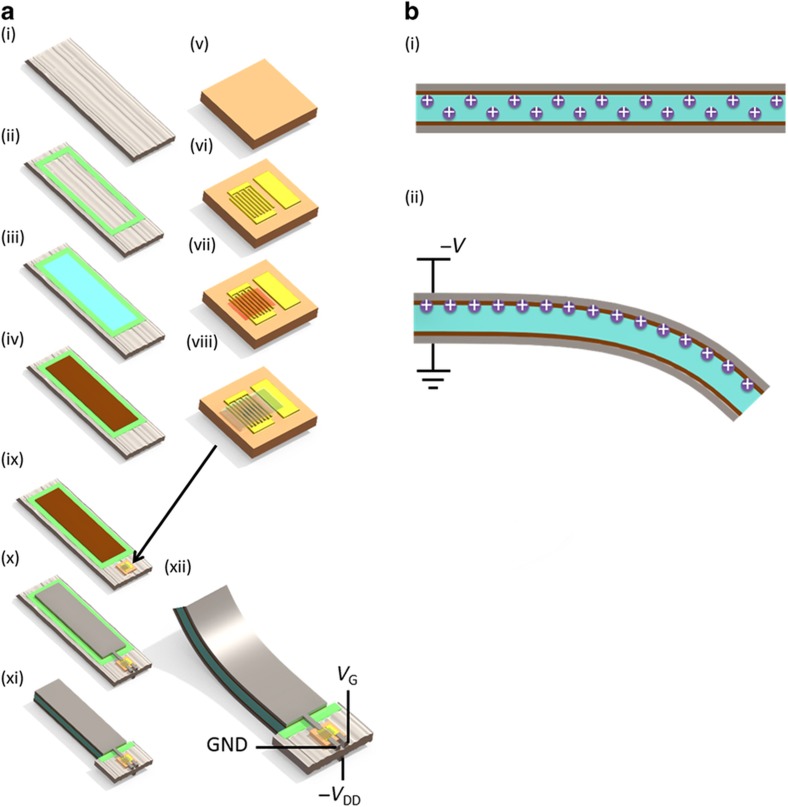
(**a**) Fabrication process flow. Actuator fabrication: (i) starting material: bare filter paper. (ii) Epoxy bank is printed into paper to prevent Nafion spreading. (iii) Nafion is printed into the paper within the bank. This step is repeated on the backside. (iv) Electrode interlayer consisting of Nafion–silver mixture is printed onto Nafion. This step is repeated on the backside. Transistor fabrication: (v) starting substrate: kapton tape. (vi) Gold source-drain and coplanar gate electrodes are evaporated through a shadow mask. (vii) Semiconductor polymer is printed onto the electrodes. (viii) Nafion gate dielectric is printed onto the semiconductor polymer layer. System integration: (ix) transistors are laminated onto the paper substrate with Nafion actuators. Actuators and transistors are immersed together in ionic liquid overnight. (x) Interconnects and actuator electrodes are printed with silver flake ink. This step is repeated on the backside. (xi) Actuators are released from surrounding paper substrate and epoxy bank to allow actuation. (xii) Voltages are applied to the system. Negative *V*_G_ is applied to the gate of the p-type transistor. Ground is connected to the source. The drain is connected to one actuator electrode. The other actuator electrode on the backside is connected to −*V*_DD_. Actuator deflection is measured. (**b**) Operating principle of the IPMC actuator. Cross-sectional view showing paper infused with Nafion in light blue and both electrode layers on either side. (i) Without any applied voltage, positive mobile ions are evenly distributed throughout the actuator. (ii) With applied voltage, positive mobile ions move toward the negative electrode, causing swelling of the actuator on that side and thus bending of the actuator.

Although much research has been conducted on printed electronics, there has been relatively little work on printed mechanical actuators^18–26^. EAPs are a promising class of materials for such printed components because they can be solution processed^27–29^. Ionic polymer–metal composites (IPMC) are particularly promising owing to the large bending strains that can be achieved at low voltages^30–34^. IPMC actuators operate by the movement of ions within a membrane that is an ionic conductor. An applied electric field forces ions to one side of the membrane causing the membrane to swell on that side, which creates a bending motion of the actuator (see Figure 1 for a schematic representation of the operating principle). IPMC actuators can be driven by low voltages below 5 V; however, they typically require large driving currents that exceed the largest currents achievable by printed organic thin film transistors (OTFTs). However, the on-current of OTFTs can be increased by orders of magnitude if an ion gel is employed as the gate dielectric^35–38^. Because of the lower operation speed of mechanical components compared with electrical circuits, the limited high-frequency performance of ion gel transistors is of little concern here. As a result, ion gel TFTs are the perfect driver for mechanical actuators whose operating principle is also based on ionic motion. We demonstrate ion gel TFTs whose gate dielectric is fabricated from the same EAP material as the IPMC actuators driven by these TFTs.

In this article, we describe the processes that were used to fabricate paper-based actuators and ion gel OTFTs. Actuators were characterized and modeled mechanically, electrically, and in terms of the electromechanical deflection. Ion gel transistors were characterized electrically to confirm that they can function as drive transistors for the actuators; such functionality was then demonstrated experimentally. Finally, a paper-based EAP actuator was manually mounted onto a paper UAV to demonstrate steering during flight. This is a first step along a promising path toward fully printed UAVs. Further work is required to create a fully integrated UAV by integrating complex control electronics, printed power sources, and other elements, such as integrating printed sensors with the printed paper-based actuators demonstrated here.

## Materials and methods

### Materials

Actuators for initial optimization were fabricated using a commercial Nafion® 117 membrane, with a thickness of 0.007 inch. Membranes were cleaned by sonication in methanol before actuator fabrication. Paper-based actuators were fabricated in Whatman®high wet strength filter paper (grade 1573). Nafion® 20 wt% solution in lower aliphatic alcohols and water was used to print both the actuators and the transistor gate dielectric. The bank material for actuator printing was Loctite®Instant Mix™ 5 Minute Epoxy. A 20 wt% dispersion of silver nanopowder (<100 nm particle size, containing poly(vinyl pyrrolidone) (PVP) as the dispersant) in a Nafion® 5 wt% solution in lower aliphatic alcohols and water was used for the actuator electrode interlayer. The second electrode layer and interconnects were printed using silver flake ink (Creative Materials 120-07). Transistors were fabricated on Kapton®tape. Interdigitated source and drain evaporation shadow masks were purchased from Ossilla. The OTFT materials lisicon®SP400 (amorphous polymer semiconductor dissolved in mesitylene and 1-methyl naphthalene) and lisicon®M001 (surface treatment for the source and drain electrodes) were provided by EMD Performance Materials Corp. (an affiliate of Merck KGaA, Darmstadt, Germany). Both actuators and transistors were immersed in the ionic liquid 1-Butyl-3-methylimidazolium tetrafluoroborate (BMI-BF4). Paper UAVs were fabricated from Boise®ASPEN® 30 copying paper. The power was supplied by a lightweight battery purchased from PowerStream Technology (GM301030, 1.4 g, 3.7 V, 62 mAh). All chemicals except for the Creative Materials ink, the epoxy, the semiconductor and the surface treatment were purchased from Sigma-Aldrich (St. Louis, MO, USA). All chemicals were used as received unless otherwise noted.

### Actuator fabrication

Paper-based actuators were fabricated by first dispenser printing an epoxy bank into the paper to limit the spreading of Nafion. The epoxy was dried for 10 min at 100 °C on a hotplate. Then, 0.2 ml cm^−^^2^ of Nafion solution was drop cast into the paper region defined by the epoxy bank and dried at 100 °C in an oven for 30 min. The drop casting and drying process was repeated on the backside. The electrode interlayer was stencil printed from a Nafion-silver nanopowder dispersion and then dried at 70 °C on a hotplate for 5 min. Silver flake ink was stencil printed as the second electrode layer and as interconnects to the transistors and the power sources. The silver flake ink was dried at 100 °C in an oven for 10 min. In between printing of the two electrode layers, the actuators were immersed in the ionic liquid overnight at 100 °C in an oven. Both electrode printing steps were performed on both sides of the actuators. Finally, actuators were cut out to release them from the epoxy bank and neighboring paper without EAP.

### Transistor fabrication

First, 75 nm of gold was thermally evaporated onto kapton tape through a shadow mask to define the interdigitated source and drain electrodes and the coplanar gate electrode^35,39,40^. The channel length and width were 20 μm and 17 mm, respectively. Electrode adhesion was improved by using a short air plasma treatment (50 W, 30 s) and applying a chromium adhesion layer (5 nm thickness). Before printing the semiconductor layer, a self-assembled monolayer surface treatment was applied to improve the contact resistance. Lisicon® M001 was applied by drop casting. After drying, the samples were rinsed with isopropyl alcohol, dried again and then dried at 100 °C for 1 min. The amorphous polymer semiconductor was patterned by stencil printing and dried at 100 °C for 5 min on a hotplate. The Nafion gate dielectric was also patterned by stencil printing and then dried at 100 °C for 5 min on a hotplate. The transistors were immersed in the ionic liquid under the same conditions as the actuators. Transistors on kapton were laminated onto the empty paper substrate adjacent to actuators before stencil printing of the interconnects.

See Figure 1 for illustrations of the processes used to produce the actuators and transistors.

### UAV integration

**Figure 2:**
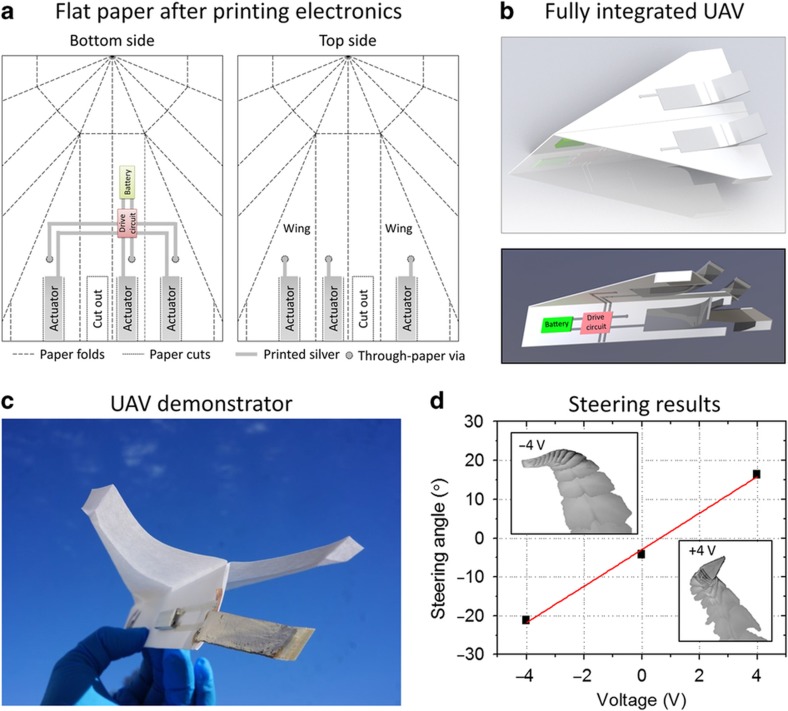
Vision of UAV integration with electronics for a fully printed UAV. (**a**) All UAV components could potentially be printed directly onto or into a flat paper substrate using roll-to-roll processes, including actuators, power sources, drive circuits, and interconnects. Printing must be performed on both sides of the paper substrate to drive both the actuator electrodes and other potential payload circuits. Connections can be made through the thickness of the paper using laser-cut through-paper vias filled with silver ink. (**b**) The final three-dimensional UAV structure would be created by folding the initially flat paper substrate. Actuators would be released by laser cutting the surrounding paper and would bend when a voltage is applied. The locations of these folds and cuts are indicated in **a**. (**c**) Paper UAV demonstrator with a separately fabricated and attached single paper-based rudder for horizontal steering and a lightweight battery connected by stencil-printed silver interconnects. (**d**) The horizontal steering angle of the demonstrator UAV depends on the applied actuator voltage, as expected, thus allowing steering to the left and right, that is, control of the yaw angle. Insets show the extracted UAV images from successive video frames overlaid on top of each other as the UAV is flying away from the camera. One can clearly see the UAV turning left and right depending on the polarity of the applied voltage (negative and positive, respectively). UAV, unmanned aerial vehicle.

Paper UAVs were folded with a folded-back nose and winglets for added stability (see Figure 2). Paper-based actuators were attached to the back of the UAV for horizontal and vertical steering, and a lightweight battery was attached to the lower base of the UAV as a power supply. The two were connected by stencil printed silver.

### Characterization

Videos of the actuator deflection response and the flight path of paper UAVs were captured using a Sony NEX-5N camera and analyzed using custom-built Matlab programs (Natick, MA, USA). The DC electrical response of the actuators and transistors and the quasi-static low-frequency capacitance were measured using an Agilent 4156C semiconductor parameter analyzer. The transistors were measured in a nitrogen atmosphere. The AC response of the actuators was measured by driving actuators using an HP 33120A function generator. The capacitance was measured using an Agilent E4980A LCR meter (Keysight, Santa Rosa, CA, USA). The mechanical stiffness of the actuators was determined by measuring the force-displacement curve under an applied tip load using a Nordson Dage 4000 Multipurpose Bondtester (Aylesbury, UK).

## Results

### Actuator integration with a paper UAV

We use printing-based fabrication to realize actuators integrated into flat paper substrates. In the future, such actuators can potentially be printed directly into large sheets of paper that are subsequently cut and folded to form the final glider structure. Thus, the flat, sheet-type nature of paper substrates offers the potential for very-high throughput, inexpensive roll-to-roll manufacturing using printing techniques. Figure 2a shows a sample layout of a future fully integrated UAV that incorporates actuators infused into paper, printed circuits, a printed power source and printed interconnects. Because printing is an entirely additive process, diverse types of devices could be integrated on the same substrate much more easily via printing than via traditional fabrication methods. All of these printing steps could be performed on large-area paper sheets. The final three-dimensional UAV structure could be created via the simple cutting and folding of the paper (Figure 2b). Different steering elements could be fabricated in this manner to control all three axes of motion: yaw, roll, and pitch. To demonstrate that paper-based IPMC actuators do indeed offer a promising route toward aerodynamic steering, we fabricated a steering element infused into paper using our actuator process and then manually mounted it on a paper UAV. The power source was an extremely lightweight battery (1.4 g) to preserve the lightweight nature of the paper substrate. In the future, the battery could be directly printed into the paper to further reduce both the weight and the cost^13^. Interconnects were directly printed onto the paper using a silver flake ink. The actuator was mounted vertically on the back of the UAV base (Figure 2c). When actuated electrically, it thus acts as a rudder that controls the yaw of the UAV. We recorded the UAV flight path and thus confirmed that our paper-based actuator acts as a rudder.

Before performing the experiments, we modeled the mechanical response of our actuators to the air drag experienced during flight. Beam equations were solved recursively to find the actuator deflection when the applied electrical force was balanced by the air drag and lift forces. The drag and lift coefficients were approximated by assuming that the actuator geometry corresponds to a flat plate at an angle to the horizontal^41^. Supplementary Figure S1 shows the calculated tip deflection at different UAV speeds. The deflection was compared with that of a commercial membrane as a reference. As the air speed increases, the tip deflection decreases due to increased aerodynamic forces. The paper-based actuator exhibits slightly lower free tip deflection at zero air velocity than does the actuator based on the commercial membrane because the former has greater stiffness. However, this increased stiffness combined with a larger electrical force results in a more stable deflection with increased air velocity, which makes this particularly advantageous for flight control. Finally, we measured the flight path of a paper UAV with an attached paper-based actuator as a function of applied voltage. Two different voltages as well as zero applied voltage were tested to demonstrate the concept. Finer control could be achieved by changing the duty cycle or the magnitude of the applied voltage (see Supplementary Figure S2 for actuator deflection as a function of voltage). The horizontal turning angle during flight clearly shows the expected trend with applied voltage allowing steering to the left and right, that is, control of yaw (Figure 2d). Videos of representative flight paths are available online as Supplementary Material. The influence over the vertical steering angle, that is, pitch, was also demonstrated by using actuators attached horizontally to the back of the glider wings, that is, perpendicular, to the direction of the actuators controlling yaw (Supplementary Figure S3). Thus, we have demonstrated that paper-based IPMC actuators can be used to influence the steering angle of glider UAVs.

The key to achieving this excellent steering performance is a robust actuator fabrication process. Here, we demonstrate the fabrication process to create fully printed actuators that are printed directly into paper. We characterize actuators both mechanically and electrically. Simple models are developed that will be important in guiding design choices when full UAV systems are integrated. The most important electrical component for creating fully integrated actuator systems is the drive transistor, which charges and discharges the EAP actuators. Because IPMC actuators require large currents but operate at relatively low frequencies, ion gel-gated OTFTs are demonstrated that use a Nafion gate dielectric to achieve large on-currents while still being printable with low-processing temperatures. The successful integration of ion gel-gated transistors with paper-based actuators is demonstrated.

### EAP actuators

#### Actuator fabrication

The use of actuators for flight control places significant demands on the mechanical performance of the actuators. We use a novel structure, in which the actuator is infused into the paper itself by exploiting the absorption of the paper. To maximize the electromechanical performance, the surface of the actuation electrode is infused into the surface of the paper and filled with EAP to form a high surface area contact, thus maximizing the specific capacitance. The actuators thus consist of three layers: the active EAP material, the infused electrode interlayer, and an overlaying ohmic contact electrode layer. The full device structure and fabrication process are shown in Figure 1. The active EAP material Nafion is printed into paper to create electromechanically active paper. It is important for the Nafion solution to fully fill the paper pores. If the ink volume is too small, then the actuator bending response will be reduced. However, it is challenging to print large amounts of liquid ink into paper because the ink will spread laterally, thereby reducing thickness and pattern fidelity. In addition, the ink viscosity must be low enough to allow complete filling of the paper pores; however, this low viscosity also promotes lateral spreading. To solve this issue, we employ a bank printed with high-viscosity epoxy. This bank prevents the lateral spreading of the relatively low-viscosity Nafion ink and enables the printing of a thick Nafion layer into the paper. After fabrication, the actuator can be released from the epoxy bank via mechanical or laser cutting. The infused electrode layer is an interlayer printed from a dispersion of silver nanopowder in a Nafion solution^42^. The distributed silver particles inside the Nafion matrix increase the effective electrode surface area and, thus, the capacitance and actuator deflection. Finally, a printed silver flake electrode is used to increase the conductivity of the electrode. By printing both electrode layers, the electrode can be patterned easily to match the specific structural requirement of the UAV glider. Before printing of the final electrode layer, the actuator is immersed in an ionic liquid (BMI-BF4), which provides the ions necessary for actuation. Because of the low vapor pressure of the ionic liquid, there are no drying-related degradation effects, which is a common problem for IPMC actuators hydrated with water-based solutions^43,44^. The stability and achievable mechanical deflection of the actuator enable the use of this device to influence the glider flight path.

#### Actuator electromechanical characterization

**Figure 3:**
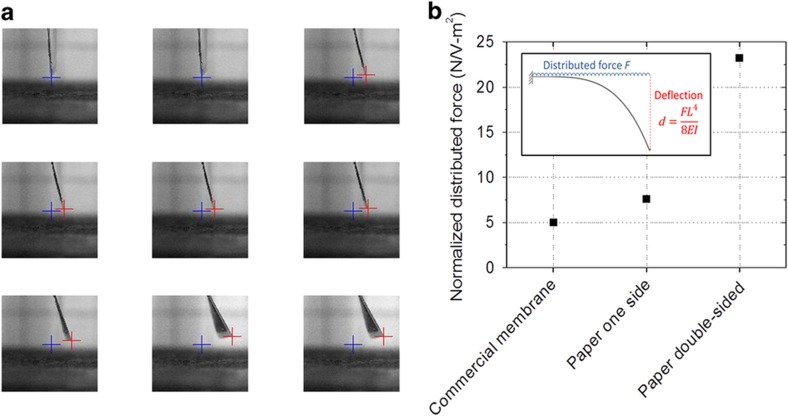
Mechanical characterization of the actuators. (**a**) Frames from a video of the deflecting actuator. The actuator is mounted as a cantilever with its tip entering the image from the top. The tip deflection is tracked by an automatic image processing algorithm. The blue cross signifies the original tip location, and the red crosses signify instantaneous tip location. The time between frames is 25 s. A constant voltage of 2 V was applied. (**b**) The distributed force created by the applied voltage *F* is calculated using beam theory for a cantilever with deflection *d*, length *L* and stiffness *EI*. Solution processed actuators with Nafion infused into paper from one side exhibit a distributed force on-par with actuators fabricated from a commercial Nafion membrane. Double-sided Nafion infused into paper significantly increases the force.

The most important performance characteristic of these actuators is their deflection response under an applied electrical bias, as shown in Figure 3a. To make design choices, a simple model is required to predict the mechanical actuator response to an applied electrical bias. We model the actuator as a cantilever with an applied distributed force due to the applied voltage^45^. The free tip deflection of the actuator without any other applied load can be calculated using beam theory:

(1)d=FL48EI

where *d* is the free tip deflection, *F* is the distributed force per unit length due to the applied voltage, *L* is the actuator length, *E* is the Young’s modulus of the actuator and *I* is the second moment of area of the actuator. We verified that the free tip deflection is proportional to the fourth power of the actuator length (Supplementary Figure S4). The tip deflection depends linearly on the applied voltage, that is, the distributed force is linearly dependent on applied voltage (Supplementary Figure S2). To calculate the distributed force created by the applied voltage, the stiffness of the actuator *EI* must be known. We measured the actuator stiffness independently by applying a point force to the tip of the actuator and fitting the following beam equation:

(2)d=FL33EI

where *F* is the applied tip force. With *EI* known (Supplementary Figure S5), the distributed force that an actuator generates can be calculated (Figure 3b). Knowledge of this electrical force enables the designer to determine the optimum geometry for a particular application, for example, trading off stiffness and free deflection. We compared the electrical force created in our novel paper-based actuators with the force created in actuators based on a commercial Nafion membrane. All other fabrication steps were the same. The paper-based actuators exhibited slightly superior performance to the commercial membrane-based actuators. These results indicate the viability of our patterned paper-based actuator process. Indeed, its performance can be improved even further by applying the EAP solution to both sides of the paper. Using this double-sided approach, the paper pores are filled more completely, and the volume of the active EAP material relative to the inactive paper fibers is increased. Thus, double-sided actuators were used for the following integration with transistors and to steer a paper UAV.

#### Actuator electrical characterization

**Figure 4:**
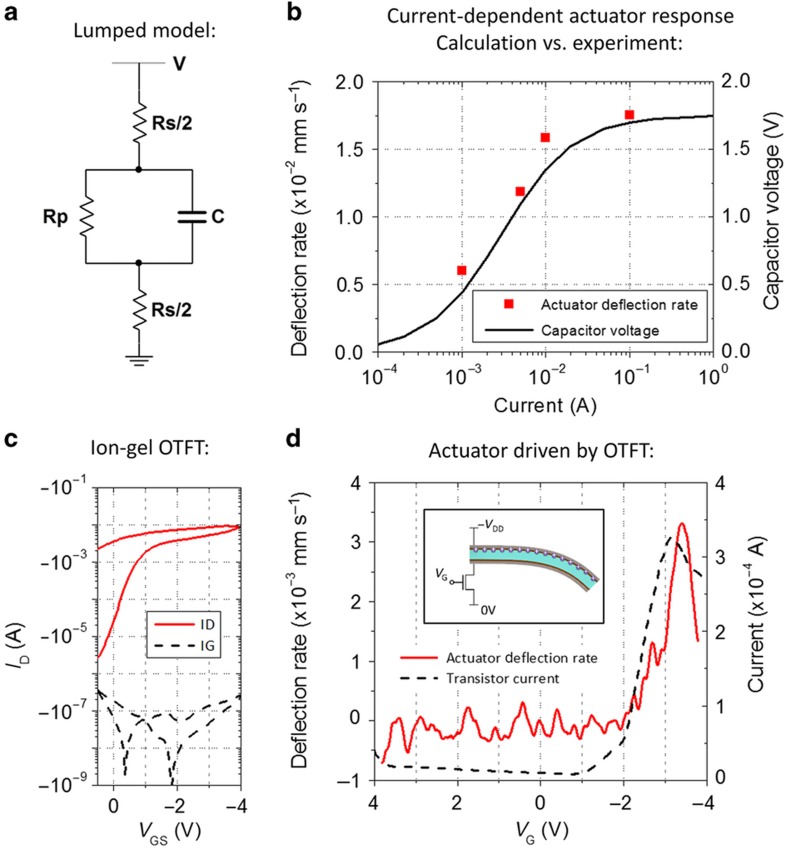
(**a**) Lumped element model describing the electrical characteristics of the EAP actuators. Charge on the capacitor C represents ionic charge responsible for the actuator deflection. Rp and Rs are parasitic resistances that reduce the charge and voltage on the capacitor. (**b**) Calculated final voltage on the capacitor in the lumped model as a function of the transistor on-current. With increasing transistor on-current, the capacitor voltage increases because the transistor channel resistance ceases to be the limiting factor. For high transistor currents, the actuator response becomes limited by the actuator’s parasitic resistances and is independent of the drive current. The measured actuator deflection rate follows the same trend for different supply current limits, confirming the importance of the drive current up to the point where deflection saturates. The applied voltage was in both cases 2 V. (**c**) The large capacitance of the Nafion gate dielectric enables very large on-currents on the order of 10 mA for printed ion gel OTFTs. *V*_DS_=−2 V. *W*/*L*=836.5. (**d**) These high-current OTFTs can drive an EAP actuator. The actuator deflection rate corresponds to the transistor on-current when they are connected in series. Toward the end of the sweep, the current and deflection rate both decrease as the actuator becomes fully charged. The supply voltage across the actuator and the transistor channel in series was 2 V. EAP, electroactive polymer; OTFT, organic thin film transistors.

To design the driving circuitry for these electromechanical actuators, they must be described by an electrical model. Different electrical models have been proposed to describe EAP actuators^46,47^. Here, we employ a simple lumped element model to capture the main characteristics of these actuators and facilitate the design process. The ionic motion responsible for the mechanical actuation is represented by a capacitance. The charging of this capacitance is inhibited by shunt leakage paths and the series resistance of the electrodes; these two non-idealities are modeled by a parallel and a series resistor, respectively (see Figure 4a for circuit diagram). The simplicity of this model allows the measured current characteristics to be fitted to analytical solutions and the extraction of the model parameters (see Supplementary Figure S6 for fit). These extracted parameters can provide insight into the differences in electromechanical performance between the different actuator implementations described above (see Figure 3b for electromechanical performance and Supplementary Figure S7 for lumped element parameters). The actuator capacitance of our paper-based actuators is significantly higher compared with that of the commercial membrane because of the increased surface area offered by rough paper. However, the single-sided paper actuator also exhibits increased series resistance and decreased parallel resistance. This explains why its electromechanical performance is not significantly improved over the commercial membrane, despite its larger specific capacitance. This is remedied by applying a second EAP layer to the bottom side of the paper actuator, thereby improving the surface properties, lowering the series resistance and increasing the actuator thickness, ultimately leading to an increase in the parallel resistance. Double-sided paper actuators exhibit series and parallel resistances that are comparable to those of the commercial membrane while still exhibiting a large specific capacitance. This explains why double-sided paper actuators exhibit superior electromechanical performance to both commercial membranes and single-sided paper actuators.

These parameters can also be used to determine the current requirements for the transistors driving the actuators. Figure 4b shows the predicted final capacitor voltage of a typical actuator for different values of transistor on-current. At low transistor on-currents, actuator charging is limited by the drive transistor in series with the actuator. As the transistor current increases, so does the capacitor voltage. At high transistor on-currents, the charging of the actuator is no longer limited by the transistor but by actuator non-idealities; as a result, the charging becomes independent of the transistor current. This phenomenon was experimentally validated by driving an actuator with a limited current of different values. The observed deflection rate corresponds to the trend of capacitor voltage calculated using the model parameters. Thus, these actuators require drive transistors that can supply several milliamps of on-current; however, the transistor on-current does not require additional increases due to the actuator’s parasitic resistances.

### Ion gel-gated transistors

#### Transistor fabrication and characterization

Traditional OTFTs cannot supply such large currents; their typical on-currents are orders of magnitude below 1 mA. However, OTFT on-current can be boosted significantly if an ionic gate dielectric is employed. Ions form a thin double layer with a very high capacitance. Supplementary Figure S8 shows the extremely high capacitance per unit area that can be achieved with our Nafion-based membranes, which is approximately six orders of magnitude larger than the capacitance of typical polymer dielectrics. The largest downside of ion gel dielectrics for electronic applications is their limited high-frequency performance due to the slow movement of ions. This limitation is much less of a concern for mechanical applications that operate at much lower frequencies than many electronic circuits (see Supplementary Figure S9 for the actuator frequency response). Currently, the frequency response of the actuators is sufficient to guide a UAV on a long-distance flight toward its target. However, if short-term steering is required, for example, to react to sensor input or to follow radio-controlled commands, then the mechanical frequency response must be increased to be on the order of 1–10 Hz. The response times of both the actuators and the transistors must be improved in this case. Such improvements can be expected if materials are optimized, potentially replacing Nafion or the ionic liquid BMI-BF4, for both the mechanically active EAP^31,44,48^ and the transistor gate dielectric^49,50^. Ion gel transistors are a perfect match for IPMC actuators that also rely on ionic motion because such actuators have a similar frequency response to the ion gel transistors and can benefit from the large drive current supplied by ion gel transistors. We fabricated Nafion ion gel transistors with a printed organic semiconductor and a printed Nafion gate dielectric. Gold electrodes were deposited by evaporation. In the future, this process can be replaced by printing electrodes with materials such as gold nanoparticles^35^ or PEDOT:PSS^37^. Figure 4c shows the transfer characteristic of a typical Nafion OTFT. The on-current on the order of 10 mA attests to the great current driving capability with the Nafion ion gel dielectric. These transistors exhibit on-off-ratios of more than three orders of magnitude, which is sufficient to achieve mechanical actuator on-off-ratios exceeding 10^2^ (see Supplementary Figure S10 for the results of a calculation of the actuator on-off-ratio based on the lumped elements model).

#### Transistor integration with actuator

Transistors printed onto kapton tape were laminated onto the paper substrate containing the patterned Nafion actuator, and the two were connected by the stencil printing of silver interconnects. The final test structure including biasing conditions can be observed in Figure 1. The transistor gate voltage was swept as the voltage across the transistor channel and the actuator, which were connected in series, was held constant, and the resulting actuator deflection was measured. Figure 4d shows the correlation between the transistor current and the actuator deflection rate. The actuator deflection rate follows the transistor current as it increases with gate voltage. The deflection and associated transistor current saturate when the capacitor is fully charged, as expected. In other words, voltage control of the ion-gated transistors provides a promising path for setting actuator deflection in an accurate and controllable manner. Thus, we demonstrated that the application of ion gel-gated transistors is a promising path toward the integration of IPMC actuators with printed electronics as the driving circuitry.

## Discussion

This work represents the first demonstration of inexpensive, disposable, electronically controlled paper UAVs. We produced several innovations that will enable this technology. The use of printing is crucial to enable low-cost manufacturing such that large numbers of UAVs can be deployed, especially for one-time use, such as in remote regions. Paper is inherently well suited for such applications because of its low cost, lightweight and biodegradability. Here, we show that paper can be made electromechanically active, which is a key enabler for fully integrated systems. We demonstrated that printing techniques can be used to fabricate this novel electromechanically active paper based on EAPs. Driving IPMC actuators with ion gel OTFTs is the key step to make IPMC actuators compatible with printed organic electronics. The matching materials and operating principles result in complementary requirements (large current, relatively low frequency), thereby making this a very interesting combination of devices. Finally, we demonstrate that paper-based EAP steering elements can indeed influence the flight path of a paper UAV glider. Further devices, such as sensors, communications or printed power sources, must be integrated to create fully functional systems. Here, we have made a first step toward achieving this novel technology by proposing a number of technological enablers and creating a functional proof of concept.

## Conclusions

EAP actuators were verified to be able to steer a paper UAV. Paper-based actuators were fabricated by printing the EAP material Nafion into paper substrates; this process made the paper electromechanically active. All of the actuator fabrication steps were performed by the low-cost fabrication process of printing. The characterization of the mechanical, electrical, and electromechanical properties of these paper-based actuators revealed that they compare favorably with actuators fabricated from a commercial EAP membrane. Ion gel OTFTs were fabricated to drive EAP actuators with significantly higher on-current than typical OTFTs with polymer dielectrics. The OTFT dielectric consists of the same Nafion as the actuators, thus highlighting the match in operating principles and operation regimes between the devices. Finally, paper-based actuators were mounted on a paper UAV and were demonstrated to be able to influence the UAV flight path.

## Notes

Supplementary Information

Flight Video +4V

Flight Video −4V
