# Supplementary material for: Printed unmanned aerial vehicles using paper-based electroactive polymer actuators and organic ion gel transistors
Source: Microsyst Nanoeng. 2016 Aug 15;2:16032. doi: 10.1038/micronano.2016.32 (PMC6444718; doi:10.1038/micronano.2016.32)
Supplement: Supplementary Information [file micronano201632-s1.pdf]

Supplementary file

Printed unmanned aerial vehicles using paper-based electroactive polymer actuators and organic ion gel transistors

Gerd Grau[†], Elisha J Frazier and Vivek Subramanian

Microsystems & Nanoengineering (2016) **2,** 16032; doi:10.1038/micronano.2016.32; Published online: 15 August 2016

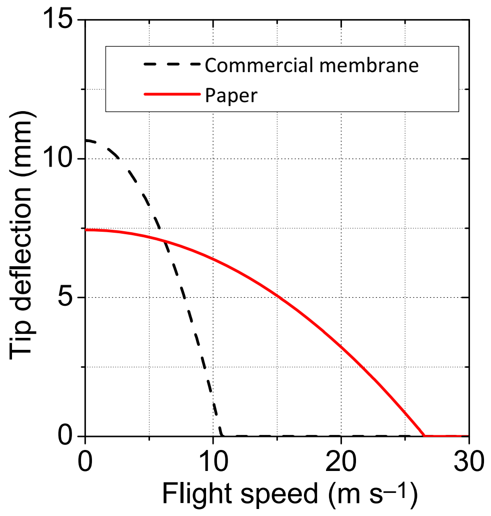

Figure S1 Calculation of actuator deflection as a function of flight speed. A larger electrical force results in larger deflection at all air speeds whilst there is a trade-off between free deflection at zero air speed and the maximum allowable air speed based on actuator stiffness. Stiffer actuators exhibit a smaller deflection at zero air speed but their performance does not drop off as fast with increasing air speed. Actuator stiffness can be modulated by careful design of actuator geometry. Paper based actuators exhibit good performance over a wide range of flight speeds due to their large electrical force as well as their large stiffness.

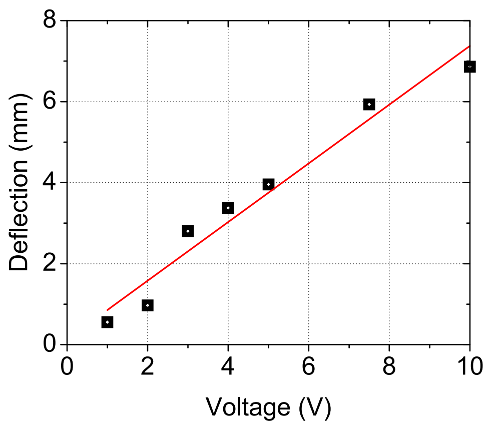

Figure S2 Measured free tip deflection and thus produced force depends linearly on applied peak voltage. Measured at 25 MHz.

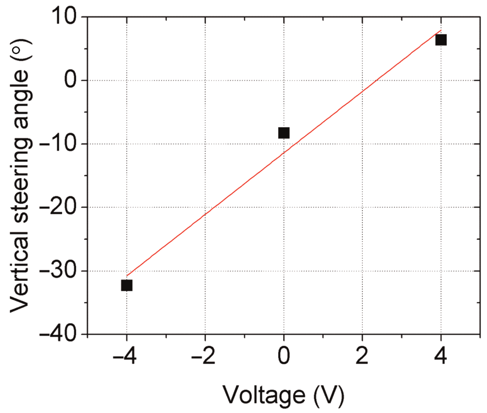

Figure S3 The vertical steering angle (pitch) can be influenced by actuators attached to the back of the wings i.e. aligned perpendicular to actuators controlling yaw. The reported angle is the initial angle of the flight path relative to the horizontal as the UAV starts its glide.

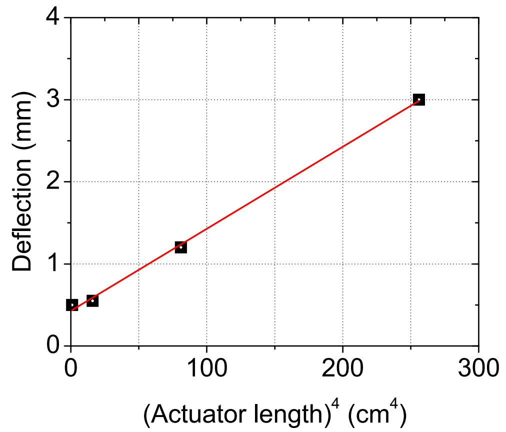

Figure S4 Measured free tip deflection of the actuator is proportional to the fourth power of actuator length. This is in agreement with our analysis based on cantilever beam theory.

Department of Electrical Engineering and Computer Sciences, University of California, Berkeley, CA 94720-1770, USA
Correspondence: Gerd Grau (grau@eecs.berkeley.edu)
[†]Present address: Department of Electrical Engineering & Computer Science, York University, 4700 Keele St., Toronto, ON M3J 1P3, Canada

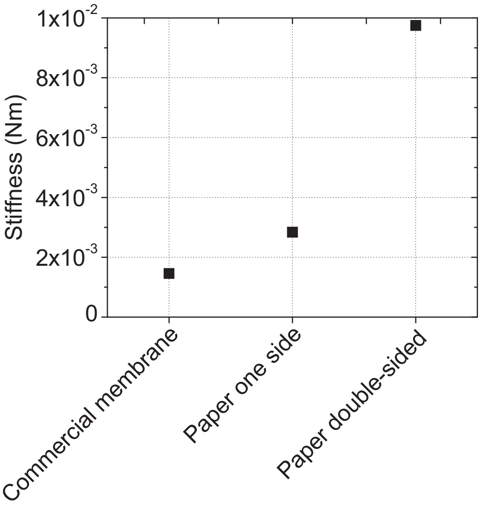

Figure S5 Measured actuator stiffness *EI*, where *E* is the Young's modulus and *I* is the second moment of area, normalized by the actuator width.

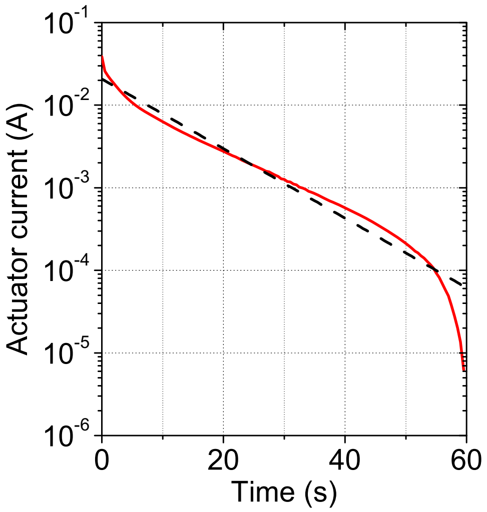

Figure S6 Fit of lumped element model to measured *I*-*t*-curve after subtracting constant current.

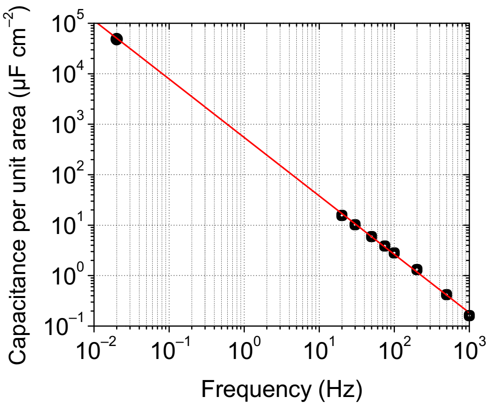

Figure S8 The capacitance per unit area for a Nafion membrane after immersion in ionic liquid is orders of magnitude larger than typical capacitances for polymer dielectrics, which are on the order of 10^{-2}μF cm^{-2}. The low-frequency capacitance (circle) was measured quasi-statically.

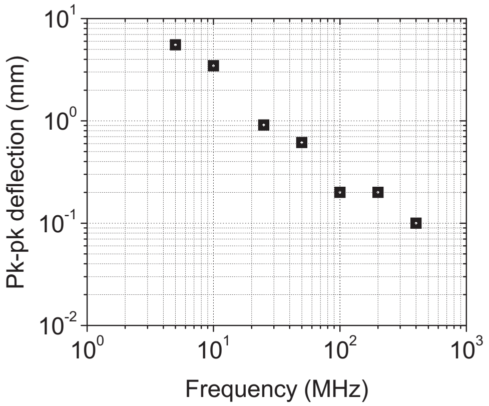

Figure S9 Measured frequency response of actuator deflection with applied square wave of $2V_{pk}$.

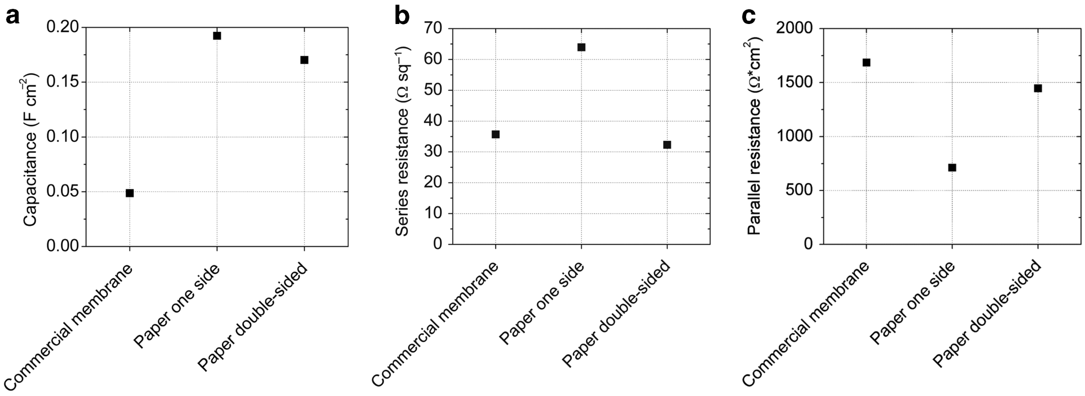

Figure S7 Lumped element parameters extracted for actuators based on a commercial Nafion membrane, Nafion infused into paper from one side and Nafion infused into paper from both sides. The differences in lumped element parameters explain the observed differences in electromechanical performance.

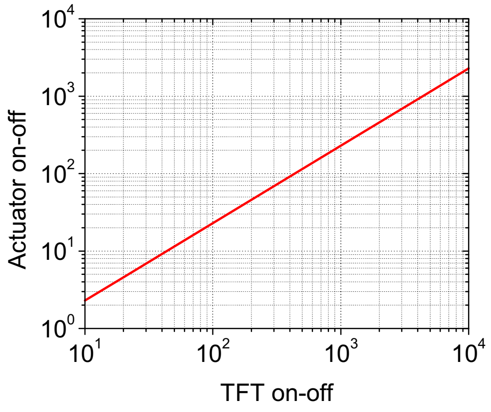

Figure S10 Calculated actuator voltage on-off-ratio as a function of drive transistor on-off-current based on lumped elements model.
